# Community evolution and phylogenetic structure of seed plants in Gansu, China

**DOI:** 10.3389/fpls.2025.1693400

**Published:** 2025-12-18

**Authors:** Qing Tian, Zizhen Li, Xiaolei Zhou, Weibo Du, Lingling Song, Rong Huang, Jia Wei

**Affiliations:** 1 Institute of Soil Fertilizer and Water-saving Agriculture, Gansu Academy of Agricultural Sciences, Lanzhou, China; 2 College of Forestry, Gansu Agricultural University, Lanzhou, China; 3 Research Institute of Forestry, Chinese Academy of Forestry, Beijing, China

**Keywords:** community assembly, environmental filtering, Gansu province, phylogenetic, spatio-temporal pattern

## Abstract

**Background:**

Understanding plant diversity is essential for revealing how species adapt and evolve across heterogeneous environments.

**Objectives:**

By integrating phylogenetics and ecology, this study aims to investigate species origins, evolutionary patterns, and biodiversity mechanisms of seed plants in Gansu Province, China. Methods: Using distribution data of seed plants, we analyzed spatial patterns, phylogenetic diversity, community structure, and floristic relationships.

**Results:**

The seed plant flora of Gansu has diverse temporal origins: 53.85% post-Miocene (0.3–23 Ma), 38.11% Paleogene (23.03–66 Ma), and 8.01% pre-Cretaceous (>66 Ma). The median diversification time is 19.61 Ma; the mean divergence time (MDT) is 28.17 ±25.65 Ma. Species richness (SR) and phylogenetic diversity (PD) are strongly correlated (P < 0.001), both showing a southeast-high to northwest-low pattern. SES-PD reveals phylogenetic overdispersion in 66 counties, suggesting migration corridors, while 14 southeastern counties show clustering due to environmental filtering. NRI and NTI analyses indicate competitive exclusion in 13 counties and environmental filtering in 55.

**Conclusion:**

This study highlights the evolutionary complexity and spatial heterogeneity of Gansu’s plant diversity, offering insights into the ecological and historical drivers shaping plant community assembly in arid and montane regions.

## 1 Introduction

The species composition within a given geographical region is the cumulative outcome of historical processes, including speciation, extinction, migration, and continuous ecological interactions (Liu et al., 2022; Tietje et al., 2023). Various landscape features differentially influence the spatiotemporal patterns of biodiversity and the processes underlying community assembly, with each process contributing to varying extents depending on the context. In some regions, the current pattern of biodiversity is attributed to an increased rate of recent speciation, effectively forming a “biodiversity cradle.” Alternatively, the origins of biodiversity might be traced back to the retention of several ancient lineages, which together constitute a “biodiversity museum” (Lu et al., 2018). Notably, the processes that generate species are not mutually exclusive with the persistence of ancient lineages, allowing some regions to simultaneously exhibit the dual characteristics of both a cradle and a museum of biodiversity (Lu et al., 2018).

The evolutionary history of regional floras is often investigated by focusing on exemplar taxa (Mishler et al., 2014) or by examining entire floristic assemblages across various taxonomic levels (Verboom et al., 2009). These studies have highlighted the significant impact of historical factors—such as geological history, climate change, and evolutionary processes—on the modern spatial patterns of biodiversity (Svenning et al., 2015), thereby providing a scientific foundation for informed biodiversity conservation decisions.

Phylogenetic research has substantially deepened our understanding of species evolutionary relationships, offering critical insights into the composition, origins, and evolutionary dynamics of biodiversity (Group, 2016). This knowledge forms the scientific basis for a more comprehensive understanding of biodiversity’s spatial distribution, as well as for the effective conservation and sustainable management of biological resources (Ci and Li, 2017). As an important metric for assessing biodiversity, phylogenetic diversity (PD) integrates evolutionary history among species, thereby surpassing traditional measures that rely solely on species counts (Crozier, 1997). PD plays a pivotal role in ecology, conservation biology, and evolutionary biology (Cadotte et al., 2008) by helping to preserve species and lineages with unique evolutionary histories, ensuring the retention of evolutionary potential, and, due to the higher functional complementarity among distantly related species, enhancing ecosystem productivity and stability (Cadotte et al., 2008). Furthermore, PD provides a robust framework for understanding ecological processes such as species coexistence, environmental filtering, and competitive exclusion (Webb et al., 2002). Studies of the phylogenetic structure of plant communities play a key role in botany, ecology, and evolutionary biology. Phylogenetic analyses clarify interspecific relationships and evolutionary histories, while also enabling researchers to pinpoint divergence nodes and major events that shaped lineage evolution. The branching patterns of phylogenetic trees, for instance, can reveal when ancient and recent clades split, identify rapid radiations, and show how innovations in traits—such as pollination modes, growth forms, or stress-tolerance strategies—have driven lineage expansion and diversification. These insights deepen our understanding of community assembly, ecological niche differentiation, and the historical and ecological drivers of large-scale plant diversity patterns (Soltis and Soltis, 2003), and thereby improve taxonomic classification, bolster biodiversity conservation, assess ecosystem stability, and promote the sustainable utilization of plant resources (Forest et al., 2007; Ye et al., 2019). With advances in molecular biology, phylogenetic studies based on genomic data have provided novel insights into the origins and diversification of plant life.

However, research on plant diversity in Gansu remains limited, particularly regarding its biogeographic characteristics and phylogenetic dimensions. Comprehensive analyses of phylogenetic diversity, evolutionary history, and the origins and biogeographic patterns of the Gansu flora are still lacking. In this study, we compiled extensive plant datasets to assess the current status of plant diversity in the region. Specifically, we aimed to: (1) clarify the evolutionary history of the regional species pool, (2) characterize the phylogenetic structure of seed plants in Gansu, and (3) identify the key factors shaping plant diversity at a regional scale. A deeper understanding of regional plant diversity patterns can provide an essential scientific basis for developing more effective biodiversity conservation strategies.

## 2 Materials and methods

### 2.1 Study area

**Figure 1:**
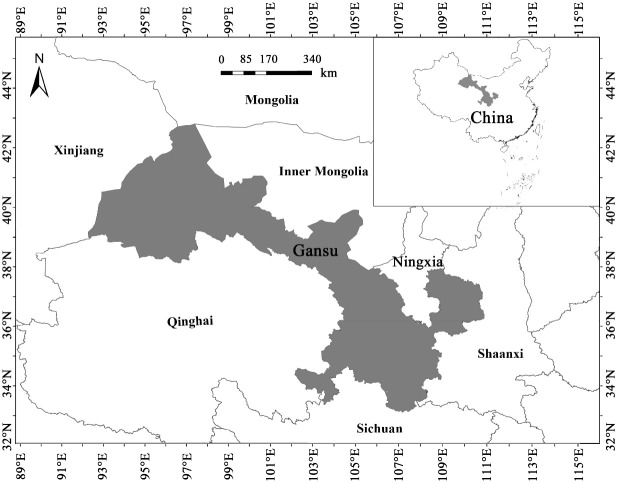
Geographical location of Gansu Province, China.

Gansu Province, a narrow and elongated region in northwestern China, spans an area of 453,700 km² between 32°11′N–42°57′N and 92°13′E–108°46′E, with elevations ranging from 526 to 5773 m (Zhang and Zhu, 2020) (**Figure 1**). Its complex topography lies at the junction of the Loess Plateau, Inner Mongolian Plateau, and Qinghai–Tibet Plateau, contributing to highly diverse natural geography and climate (Mao et al., 2020). The region encompasses four major climatic types—including warm temperate and cold temperate continental climates (Rivas-Martínez et al., 2011)—and serves as a critical water conservation and supply zone for the upper reaches of the Yangtze and Yellow Rivers (Hu, 2024). Owing to its distinctive geological and climatic heterogeneity, Gansu represents a key biogeographic transition and intersection zone in China, overlapping the Holarctic, East Asiatic, Tethyan, and Qinghai–Tibet Plateau floristic regions (Ye et al., 2019; Li et al., 2023; Wang and Lian, 1997). This setting has fostered extremely rich plant communities with ancient floristic components distributed across wide altitudinal, climatic, and soil gradients (Li et al., 2025), thereby supporting unique floristic assemblages shaped by the region’s varied physical conditions (Hu, 2024).

### 2.2 Construction of plant dataset

From May 2021 to January 2022, we compiled records of seed plants in Gansu Province. The dataset was developed using resources such as the Flora of Gansu (Gansu Flora Committee, 2005), Lanzhou Flora (Sun, 1962), Flora of China (Committee of China Flora, 2004), Flora of Chinese Deserts (Liu, 1985), Flora of the Loess Plateau (Fu, 2000), Flora of Ziwu Mountain Woody Plants (Liu, 1998), Flora of Kongtong Mountain (Gao, 1998), Flora of Xiaolong Mountain Higher Plants in Gansu Province (An, 2002), Illustrated Guide to Gannan Trees (Feng, 1994), Gansu Medicinal Plant Resources (Zhao, 2004), and Vegetation of Gansu (Huang, 1997), along with relevant journal articles documenting all available plant records in Gansu. To supplement and refine the dataset, we consulted authoritative databases, including the Chinese Virtual Herbarium (www.cfh.ac.cn), Chinese Digital Herbarium (www.cvh.ac.cn), National Specimen Information Infrastructure (www.nsii.org.cn), Global Biodiversity Information Facility (GBIF) (www.gbif.org), Catalogue of Life China (www.sp2000.org.cn), Catalogue of Life (www.catalogueoflife.org), and the Flora of China (www.iplant.cn). These platforms provided additional plant records to enhance the completeness and accuracy of the dataset. The data were purged of duplicates and records that lacked reliable georeferencing to the level of the county. Excluding non-native species, the final matrix consisted of 5244 records belonging to 1131 genera and 170 families. The names of all species were standardized using the R (R Core Team, 2017) software package ‘plantlist’ (Zhang, 2019), with the order of families based on the nomenclature of Angiosperm Phylogeny Group IV (Group, 2016) and on the most recent gymnosperm classification system (Yang et al., 2022).

Due to limitations in the spatial accuracy and completeness of available species distribution records—many of which lack precise geographic coordinates or are based on historical or coarse-scale occurrence data—plant species information in Gansu can only be reliably resolved at the county level. The administrative boundaries within Gansu closely follow natural features like mountain ridges and river paths, making counties the most suitable minimum units for this study. This approach aligns with the geographical distribution characteristics of plants in Gansu and enhances the precision of ecological and biodiversity analyses. To ensure geographical continuity and integrity for the analysis of species composition and phylogenetic relationships, adjustments were made to merge or consolidate adjacent areas or fragmented territories within counties. Therefore, the study area was divided into 80 county-level geographical units (**Supplementary Figure S1**; **Supplementary Table S1**). The presence or absence of seed plants in each county was determined by extracting distribution information from the county grid and constructing a presence-absence matrix (**Supplementary Table S2**).

### 2.3 Phylogenetic analysis

This study constructed the phylogenetic tree for seed plants in Gansu based on the mega-tree created by Jin and Qian (2019) using the Phylomatic framework. This mega-tree integrates various phylogenetic insights: it includes the evolutionary tree of angiosperms calibrated with paleontological and fossil data, derived using penalized likelihood (Smith and O’Meara, 2012) and Bayesian relaxed clock methods (Drummond et al., 2006; Drummond and Rambaut, 2007) as per Magallón et al. (2015). It also incorporates a dated phylogenetic tree for seed plants (GenBank taxa with a backbone from the Open Tree of Life project: GBOTB) constructed by Smith and Brown (2018) using genetic and systematic data from the GenBank database (https://tree.opentreeoflife.org), along with phylogenetic branches for ferns from Zanne et al. (2014). This mega-tree comprises 10,587 genera and 74,533 species of vascular plants, covering all 479 families of vascular plants globally (Jin and Qian, 2019).

Using this mega-tree, the families identified within the study region were integrated into the existing backbone phylogeny through R software with the ‘V.PhyloMaker2’ package (Jin and Qian, 2019) to generate a phylogenetic tree at the family level. For genera that do not appear in the mega-tree, their taxonomic positions were determined according to the APG IV classification system and placed at the node corresponding to their nearest sister genus in the mega-tree (Jin and Qian, 2019). When sister relationships at the generic level were unresolved, the missing genera were arranged as multiple genera within the same family, following previously published approaches (Hardy et al., 2012). The constructed phylogenetic tree was then visualized using the online software Interactive Tree of Life (ITOL) (https://itol.embl.de) (**Supplementary Figure S2**).

### 2.4 Spatial differentiation patterns

To investigate the spatial differentiation patterns of Gansu seed plant genera, spatial distribution data were integrated with a phylogenetic tree to compute the mean divergence time (MDT) for each grid cell (Lozupone and Knight, 2005; Lu et al., 2018). The MDT was calculated as follows:


$$\text{MDT = }\frac{\sum_{i=1}^{n}(AGE_{i}{\times \text{S}}_{i})}{\sum_{i=1}^{n}S_{i}}$$

where AGE_i_ represents the divergence time of the *i*th genus within the grid cell (with *i* = 1, …, *n*), and S_i_ denotes the number of species of the *i*th genus present in that cell. The divergence time for each genus was determined based on the median crown divergence time.

To further clarify the MDT for each grid cell, a null model was employed to identify divergence hotspots that reflect both ancient and recent evolutionary events within the Gansu seed plant flora. The observed value for the null model was defined as the mean age of the youngest and oldest quartiles in each grid cell. Using the complete set of genera surveyed in Gansu as a species pool, genera were randomly reassigned to generate a null distribution of the ages for the youngest and oldest quartiles. This process yielded the standardized effect size of the mean divergence time (SES-MDT) for each grid cell, calculated as follows:


$$\text{SES}-\text{MDT}=\frac{\text{M}{\text{DT}}_{\text{observed}}\;-\;\text{M}{\text{DT}}_{\text{random}}}{\text{s}.\text{d}.(\text{M}{\text{DT}}_{\text{random}})}$$

In this equation, MDT_observed_ represents the empirically observed mean divergence time, MDT_random_ is the expected mean divergence time obtained from 999 randomizations, and s.d.(MDT_random_) is the standard deviation of the randomized expected values. According to the SES-MDT, higher values indicate a significantly greater antiquity of the flora, whereas lower values signify a more pronounced youthfulness. Specifically, an SES-MDT value less than -1.96 denotes a significant deviation toward youthfulness (*P<* 0.05), while an SES-MDT value greater than 1.96 indicates a significant bias toward antiquity (*P<* 0.05) (Lu et al., 2018). All calculations were performed using R version 4.3.2 (R Core Team, 2017).

### 2.5 Phylogenetic diversity

Phylogenetic diversity (PD) is defined as the cumulative sum of branch lengths on the phylogenetic tree for all species present within a given grid cell (Faith, 1992). To further quantify the complexity of each grid cell, the standardized effect size of phylogenetic diversity (SES-PD) is calculated as follows (Ye et al., 2019):


$$\text{SES}-\text{PD}=\frac{\text{P}{\text{D}}_{\text{observed}}\;-\;\text{P}{\text{D}}_{\text{random}}}{\text{s}.\text{d}.(\text{P}{\text{D}}_{\text{random}})}$$

In this equation, PD_observed_ represents the observed phylogenetic diversity, while PD_random_ is the expected diversity value derived from 999 randomization iterations. The term s.d.(PD_random_) denotes the standard deviation of these randomized values. The SES-PD metric allows for the comparison of observed values against random expectations, thereby revealing the phylogenetic structure within the community.

A higher SES-PD value indicates greater phylogenetic dispersion among species, which is often linked to increased community structural complexity. Conversely, a lower SES-PD value indicates that species are more closely related, resulting in a more concentrated phylogenetic structure and a simplified community overall. Specifically, an SES-PD value less than -1.96 signifies that the community complexity is significantly lower than expected (*P<* 0.05), while a value greater than 1.96 denotes significantly higher community complexity (*P<* 0.05) (Faith, 1992; Faith et al., 2004; Winter et al., 2013; Lu et al., 2018). All statistical analyses were conducted using R with the ‘ape’ package (Oksanen et al., 2013) and the ‘picante’ package (Kembel et al., 2010).

### 2.6 Phylogenetic structure of plant communities

Mean Pairwise Distance (MPD) and Mean Nearest Taxon Distance (MNTD) quantify phylogenetic relatedness within each ecological grid cell: MPD is the cumulative sum of phylogenetic distances among all species pairs, whereas MNTD sums the distance from each species to its closest phylogenetic neighbor (Webb, 2000). In analyses that do not incorporate geological history, high MPD and MNTD indicate more distantly related assemblages and are typically interpreted as evidence for competitive exclusion, whereas low MPD and MNTD indicate closer relatedness and suggest habitat filtering (Webb, 2000; Miller et al., 2017).

To further dissect community structure, the Net Relatedness Index (NRI) and Nearest Taxon Index (NTI) combine MPD and MNTD with null models (Webb et al., 2002): NRI reflects overall phylogenetic depth, NTI emphasizes nearest-neighbor structure. Together, they provide complementary insights into species coexistence mechanisms. Positive NRI/NTI denote phylogenetic clustering, significantly negative values indicate overdispersion, and values near zero indicate a random structure consistent with neutral theory; significant departures from zero imply niche-based processes (Webb et al., 2002). Specifically, NRI or NTI< -1.96 indicates significant overdispersion (P< 0.05), suggesting competitive exclusion, whereas values > 1.96 indicate significant clustering (P< 0.05), suggesting habitat filtering (Webb et al., 2002; Ritchie et al., 2021; Wiens, 2004). The indices are computed as follows:


$$\text{NRI}\;=\;-1\times \frac{\text{MP}{\text{D}}_{\text{observed}}\;-\;\text{MP}{\text{D}}_{\text{random}}}{\text{s}.\text{d}.(\text{MP}{\text{D}}_{\text{random}})}$$

$$\text{NTI}\;=\;-1\times \frac{\text{MNT}{\text{D}}_{\text{observed}}\;-\;\text{MN}{\text{TD}}_{\text{random}}}{\text{s}.\text{d}.(\text{MN}{\text{TD}}_{\text{random}})}$$

Here, MPD_observed_ and MNTD_observed_ represent the observed values of MPD and MNTD, respectively. The expected values MPD_random_ and MNTD_random_ are obtained from 999 randomization iterations, and s.d.(MPD_random_) and s.d.(MNTD_random_) denote their corresponding standard deviations.

## 3 Results

### 3.1 Origin composition

**Figure 2:**
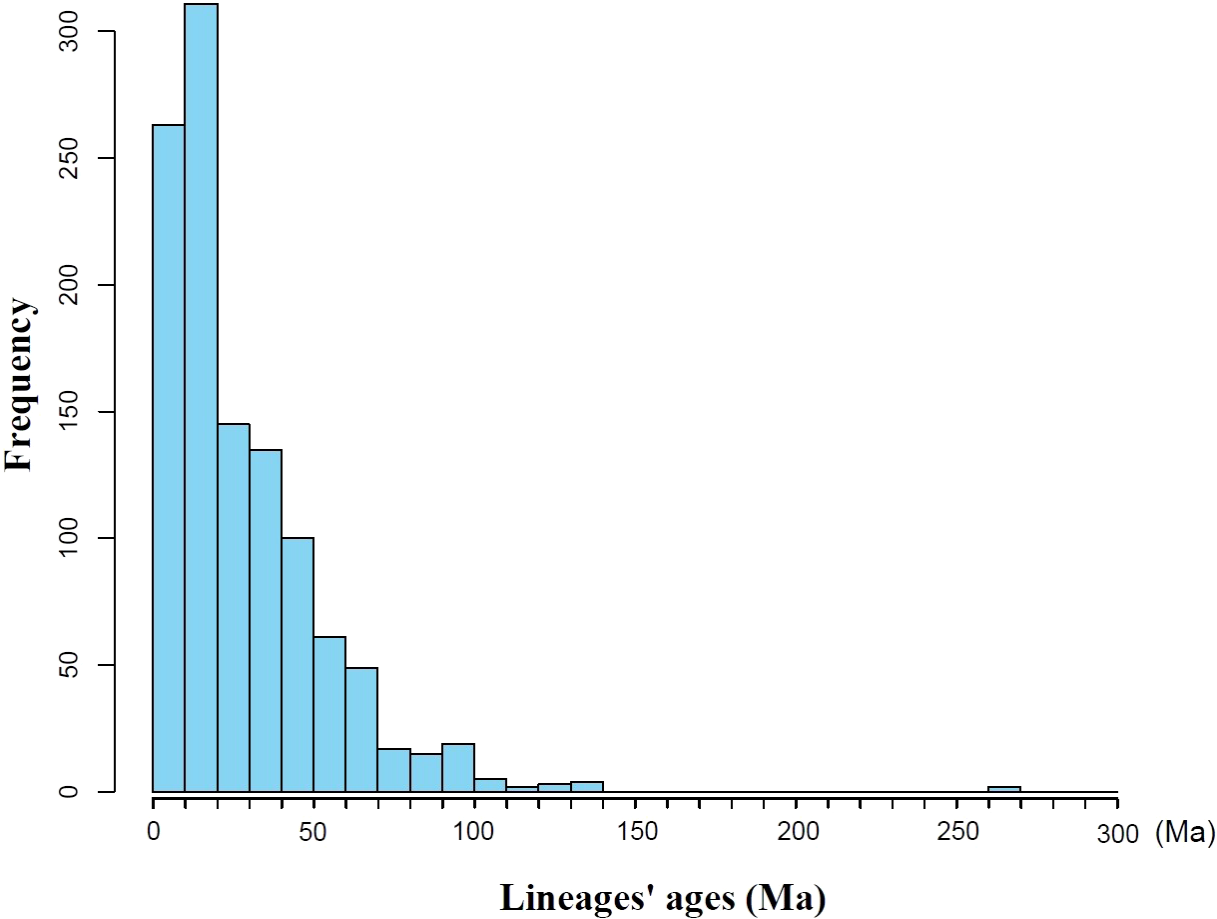
Frequency distribution of lineages’ ages of Gansu plant.

Based on the seed plant genera in Gansu, a phylogenetic tree was constructed (**Supplementary Figure S2**), and the divergence times for all genera were extracted from each clade (**Supplementary Figure S3**). Statistical analysis revealed that the mean divergence time for all clades in Gansu is 28.17 ± 25.65 Ma, with a median of 19.61 Ma. Notably, (**Figure 2**) the majority of clades (53.85%) originated less than 23.03 Ma, corresponding to the Miocene. Clades originating from the Paleogene period (66.00–23.03 Ma) account for 38.11% of all clades, while those with origins in the Cretaceous (145.00–66.00 Ma) constitute 7.89%, including genera such as *Ephedra* and *Gnetum* (Rydin et al., 2006; Ickert-Bond and Renner, 2016). The divergence times of plant groups in Gansu span a wide range, with the oldest clades dating back to the Late Permian (approximately 270 Ma), such as *Ginkgo* (Zhou and Zheng, 2003).

### 3.2 Phylogenetic origin time of the floristic assemblage

**Figure 3:**
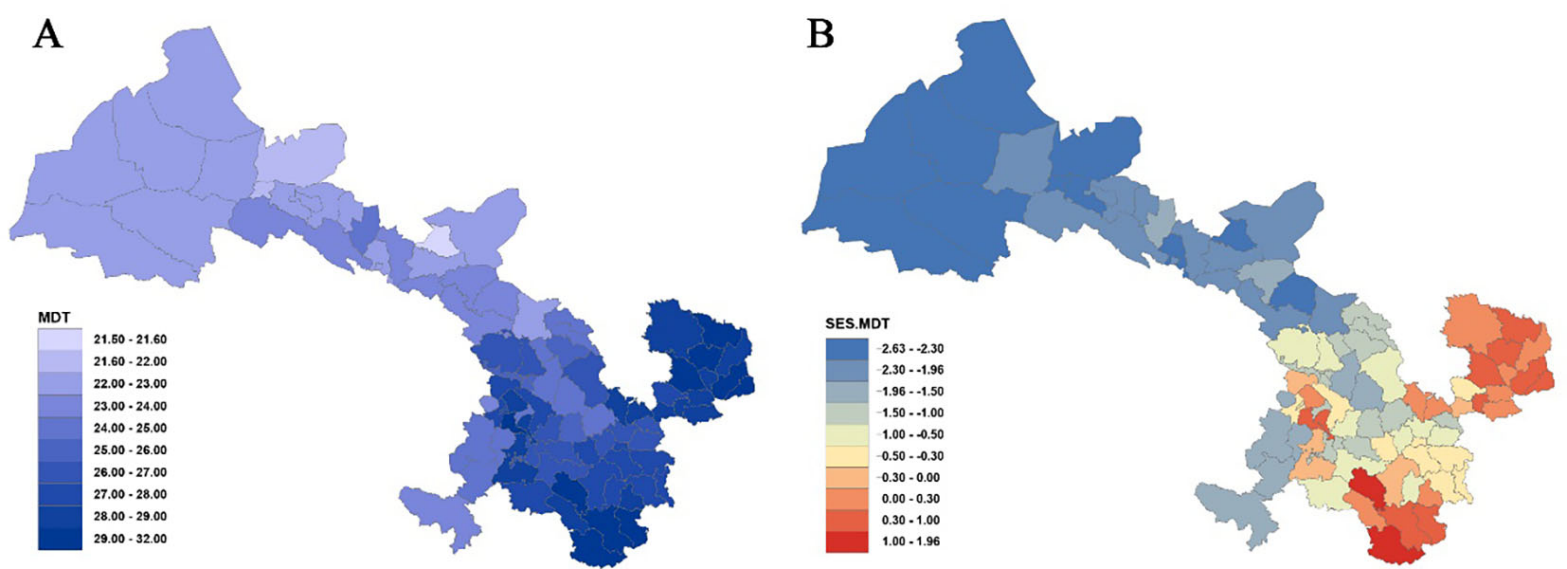
Mean differentiation time (MDT) **(A)** and standardized effect size of MDT (SES-MDT) **(B)** for Gansu County grid cells.

Statistical analysis of the mean differentiation time (MDT) across 80 county grid cells in Gansu (**Figure 3A**, **Supplementary Table S5**) indicates that the oldest MDT is observed in the grid cell of Dangchang County at approximately 31.83 Ma, while the youngest MDT is found in Jinchang County at 21.52 Ma. Overall, the regional flora predominantly dates from the late Oligocene to the early Miocene. Analysis reveals that among 22 counties with MDT values below 24 Ma, except for Maqu (23.74 Ma) and Jishishan (23.68 Ma), most are located in the Hexi region—such as Wuwei (23.99 Ma), Tianzhu (23.78 Ma), Gulang (23.38 Ma), and Shandan (23.14 Ma). For the 23 counties with MDT values between 24 Ma and 27 Ma, these are primarily situated in the Loess Plateau and parts of the Gannan Plateau, for example, Baiyin (24.45 Ma), Dingxi (24.77 Ma), Tongwei (24.97 Ma), Lanzhou (25.53 Ma), Yongdeng (26.36 Ma), Qin’an (26.64 Ma), and Gangu (26.86 Ma) in the Loess Plateau, as well as Luqu (24.11 Ma), Xiahe (24.24 Ma), and Hezuo (24.97 Ma) in the Gannan Plateau. Counties with MDT values exceeding 27 Ma are mainly found in eastern Gansu, west of the Qinling Mountains, within the Longnan mountainous region, and across much of the Gannan Plateau (e.g., the entire Longnan prefecture, parts of Tianshui, all of Qingyang, most areas of Pingliang, and a large portion of Gannan).

Furthermore, analysis of the standardized effect size of MDT (SES-MDT) (**Figure 3B**, **Supplementary Table S5**) shows that 19 counties exhibit significantly younger ages (*P<* 0.05), primarily within the Hexi Corridor, whereas pronounced antiquity is concentrated in Wen County and Dangchang.

At the provincial scale, the plant taxa in the Longnan region of Gansu display relatively older origin times, whereas the flora in the entire Hexi region is significantly younger. Overall, the origin times of plant taxa follow a stepwise gradient: as one moves westward, assemblages become progressively younger, while moving toward southeastern Gansu, assemblages become older.

### 3.3 Phylogenetic diversity

**Figure 4:**
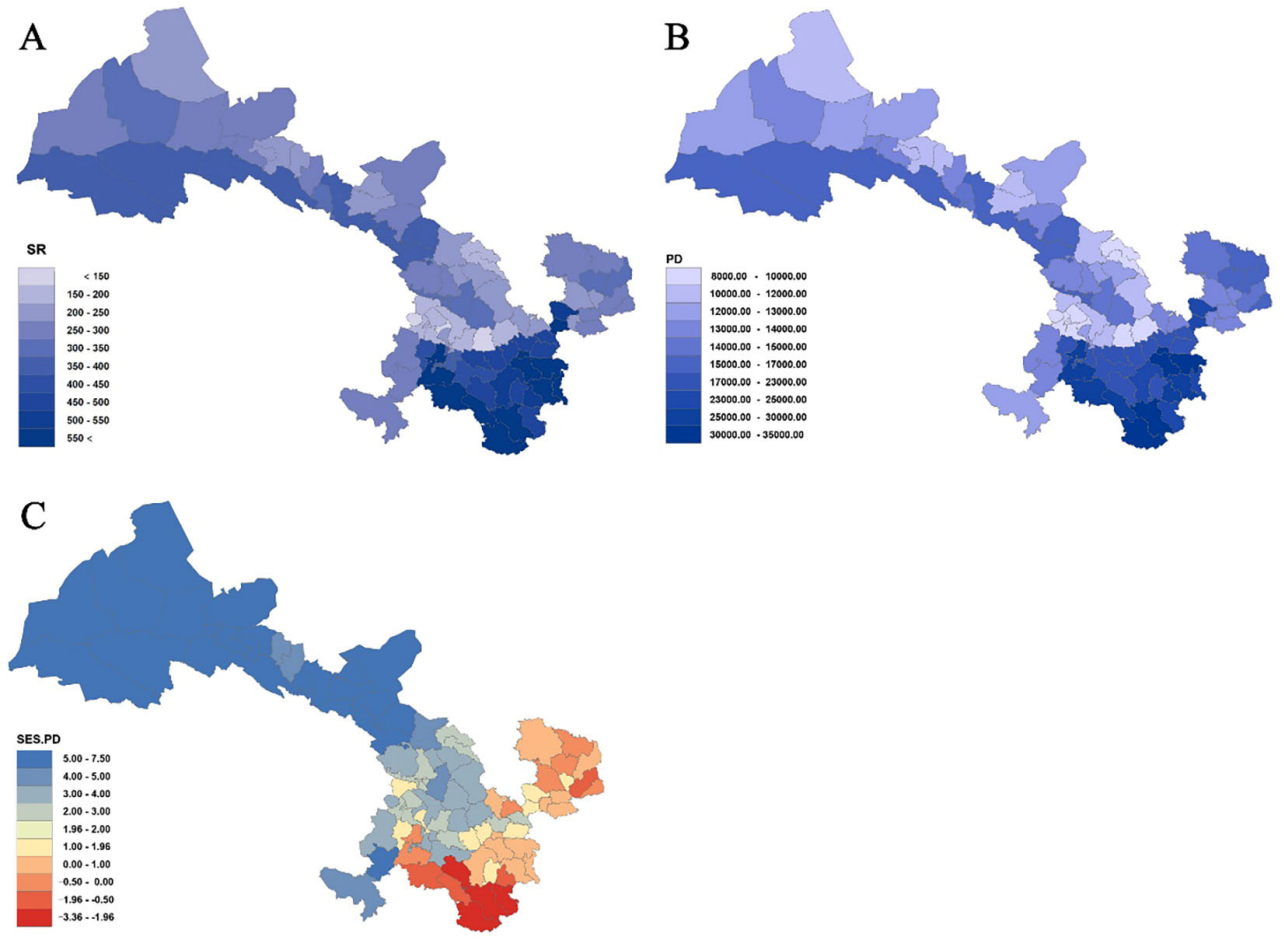
Species richness (SR) **(A)**, phylogenetic diversity (PD) **(B)**, and standardized effect size of PD (SES-PD) (C) in Gansu County grid cells.

**Figure 5:**
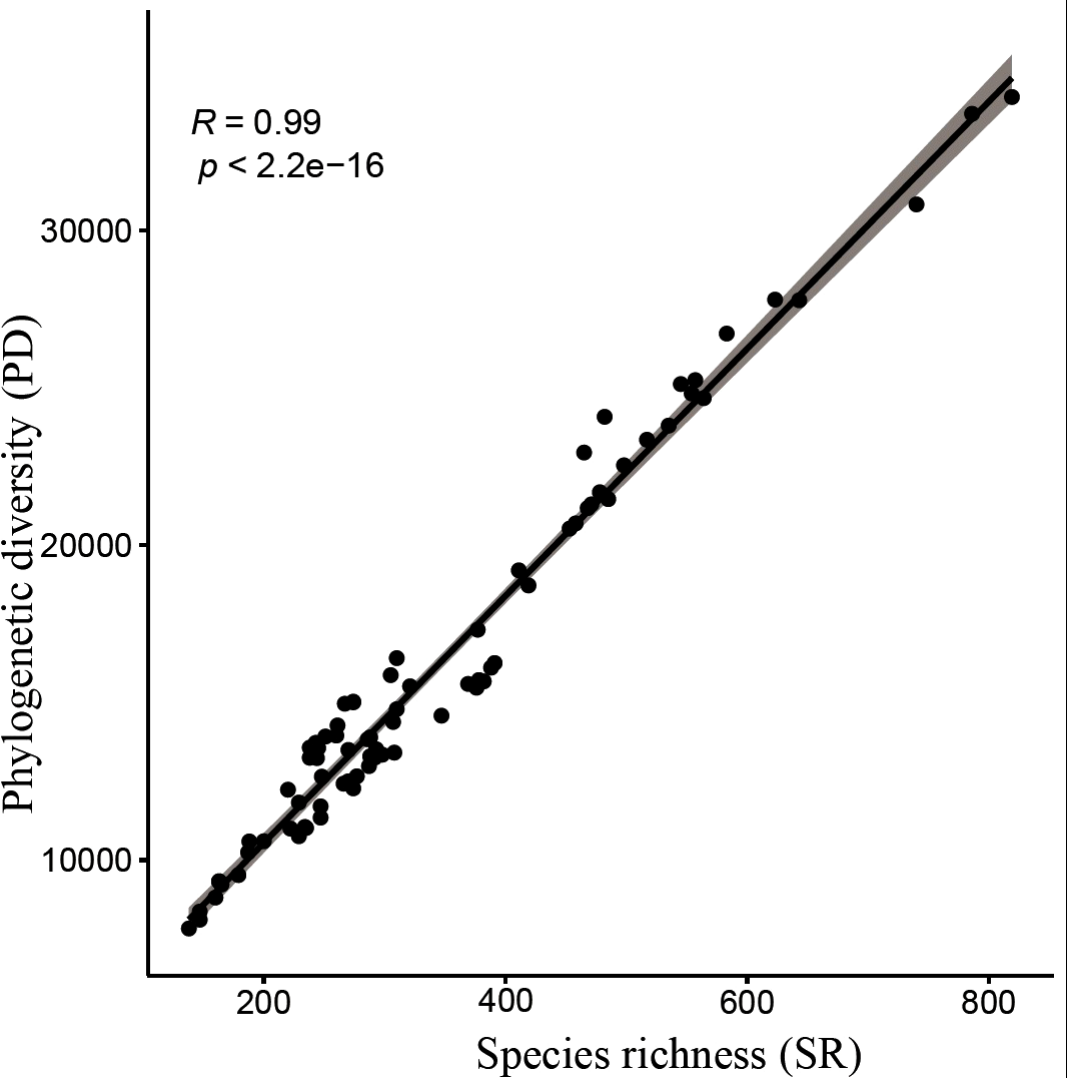
Relationship between species richness and phylogenetic diversity.

Analysis of species richness (SR) and phylogenetic diversity (PD) across 80 county grid cells (**Figures 4**, **5**; **Supplementary Table S6**) reveals a significant positive correlation between species diversity and PD (*P<* 0.001), which is consistent with previous studies (Ye et al., 2019; Cai et al., 2020). Notably, Wen County in the Longnan region exhibits the highest PD, while Guanghe in Linxia Prefecture displays the lowest PD. Overall, the spatial distribution of PD for Gansu seed plants shows a trend of higher values in the southeast and lower values in the northwest. In particular, county grid cells adjacent to the Qilian Mountains in the Hexi Corridor demonstrate higher PD compared to those in the interior of the corridor.

Further analysis of the standardized effect size of phylogenetic diversity (SES-PD) (**Figure 4C**; **Supplementary Table S6**) reveals distinct spatial patterns across the study region. Among the county grid cells examined, 66 exhibit SES-PD values greater than 0, of which 46 are statistically significant (*P<* 0.05). These are mainly distributed in the Hexi Corridor, parts of the central Gansu Loess Plateau, and areas of the Gannan Plateau. In contrast, 14 grid cells show SES-PD values below 0, with four of these (Kangxian, Wudu, Dangchang, and Wenxian) being statistically significant (*P<* 0.05). These results indicate that northwestern Gansu is predominantly characterized by phylogenetically overdispersed assemblages, whereas southeastern parts of the province tend to display phylogenetically clustered communities.

### 3.4 Phylogenetic structure

**Figure 6:**
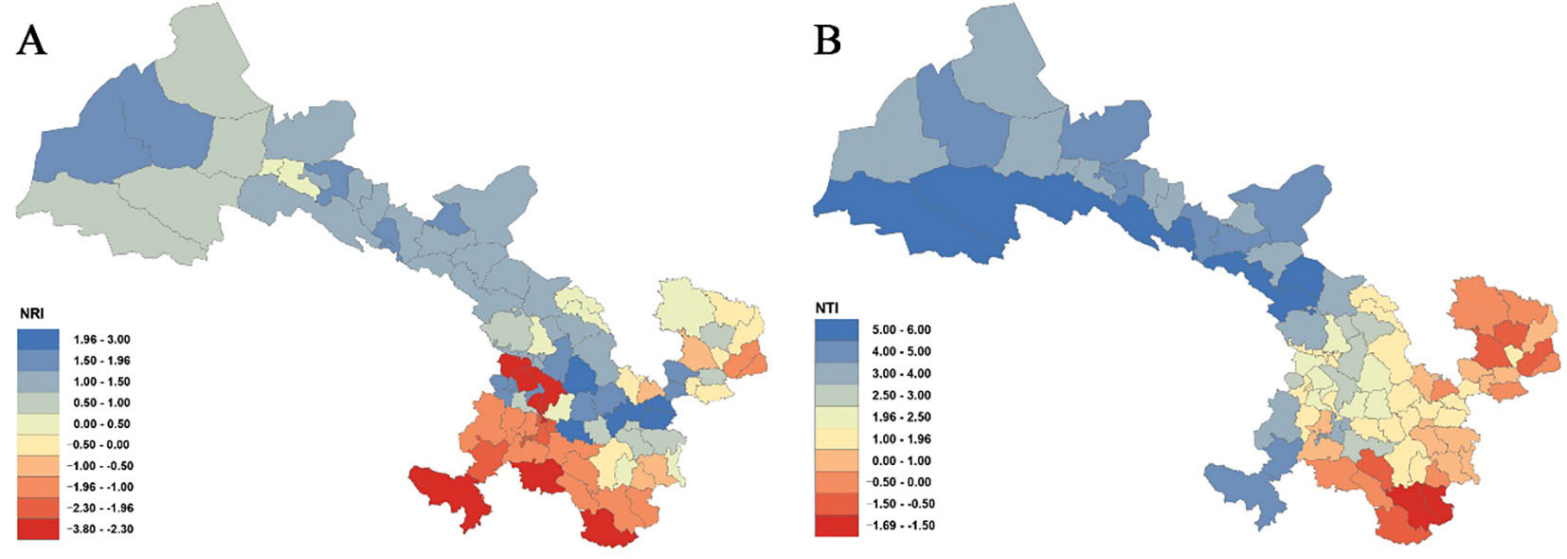
Net relatedness index (NRI) **(A)** and nearest taxon index (NTI) **(B)** of Gansu County grid cell.

Analyses of the phylogenetic structure using NRI and NTI (**Figure 6**; **Supplementary Table S6**) reveal that 57 county grid cells exhibit NRI values greater than 0, with 6 of these showing statistically significant clustering (*P<* 0.05) in counties such as Zhangjiachuan, Qin’an, Gangu, Qingshui, Zhangxian, and Anding. In contrast, 30 county grid cells have NRI values below 0, 9 of which are statistically significant (*P<* 0.05) in counties such as Kangle, Yongjing, Dongxiang, Lintao, Wen, and Diebu. Regarding NTI, 72 county grid cells present NTI values greater than 0, with 40 of these reaching significance (*P<* 0.05), mainly in counties along the Hexi Corridor as well as in parts of central Gansu (e.g., Dingxi in Longnan and some areas in Linxia). Only 15 county grid cells show NTI values less than 0, and none of these are statistically significant (*P<* 0.05).

A combined analysis of NRI and NTI (**Figure 6**; **Supplementary Table S6**) indicates that across the province, there are 2 county grid cells where NRI > 0 while NTI< 0 (e.g., Huanxian and Qingcheng); 17 grid cells exhibit NRI< 0 with NTI > 0 (e.g., Heshui, Chongxin, Jingning, Qingyang, Lixian, Huixian, Hezuo, Xiahe, and Zhuoni); 13 grid cells show both NRI and NTI< 0 (e.g., Wenxian, Diebu, Zhouqu, Wudu, and Kangxian); and 55 grid cells have both NRI and NTI > 0, predominantly distributed in the Hexi Corridor and in the Loess Plateau regions of central and western Gansu.

## 4 Discussion

### 4.1 Origin of the Gansu flora

Understanding the divergence and evolution of plants across geological periods is crucial for elucidating the processes of speciation and adaptive evolution. Numerous studies have long focused on identifying the historical geological and climatic events that have shaped the formation and evolution of the Gansu flora. Our findings indicate that the mean divergence time is 28.17 ± 25.65 Ma, with a standard deviation nearly equal to the mean, reflecting a highly skewed distribution of lineage divergence times. This high variability underscores the heterogeneous evolutionary history of the regional flora, likely driven by the presence of paleo‐relict lineages such as Ginkgo. In this context, the median origin time of plant lineages in Gansu is 19.61 Ma (early Miocene), which is notably older than the 13.60 Ma median reported for the broader East Asian flora (Chen et al., 2018). The similarity in origin times between Gansu and the Kunlun flora (19.49 Ma) (Du, 2021) further suggests that these regions may share comparable evolutionary histories and biogeographical processes.

This ancient characteristic is closely linked to Gansu’s unique geological history and geographic location. Positioned at the confluence of the Tibetan, Mongolian, and Loess Plateaus, Gansu has undergone complex geological evolution, resulting in pronounced environmental gradients and highly heterogeneous habitats that serve as critical corridors for gene flow and refugia. The uplift of the Tibetan Plateau and the aridification of the Asian interior, particularly during key periods such as the early Pleistocene, provided shelter for ancient plant lineages by creating localized microclimates and mitigating extinction pressures from global climatic events such as the Quaternary glaciations (Liu et al., 2017; Yang et al., 2006). During the Last Glacial Maximum, the vegetation in the western Loess Plateau shifted to desert steppe or sparse steppe under cold and dry conditions (Tang et al., 2007), placing extreme pressure on temperate species. However, earlier humid phases during the Middle Holocene allowed for the expansion of mixed conifer and broad-leaved deciduous forests in the region (Zhao et al., 2024), while the late Last Deglaciation and early Holocene were characterized by climate instability and temperate-dry conditions with grassland or shrub-grassland dominating the landscape (Wei et al., 2009). These recurrent oscillations between forest expansion under warm and wet conditions and grassland or desert expansion amid cold and dry conditions have generated dynamic refugia and facilitated lineage divergence. Compared to other regions in East Asia, Gansu’s diverse topography and climate have facilitated both the preservation and diversification of its flora (Favre et al., 2015), thereby contributing to the continuity of ancient plant lineages. Moreover, the presence of plant groups originating from the Paleogene and earlier indicates that this region has provided long-term refugia, aiding in the conservation of genetic diversity. The broad temporal range of lineage origins, from the Late Permian to the Neogene, further underscores Gansu’s role in enabling the coexistence and ongoing evolution of plant groups from multiple geological epochs.

### 4.2 Regional differences in origin times

Differences in the mean divergence time (MDT) of plant lineages across various regions within Gansu Province reflect the profound influence of regional geological history and climatic fluctuations on the evolution of plant diversity. Plant lineages in the Longnan region exhibit the oldest origin times, whereas those in the Hexi Corridor are comparatively younger. Overall, there is a discernible trend of progressively younger origin times from the southeast to the northwest.

The Longnan region, located in southeastern Gansu, is characterized by its complex topography and warm, humid climate, forming part of the Qinba mountain system. This area may have been less impacted by the Quaternary glaciations and could have served as a refuge for ancient plant lineages, resulting in older divergence times (Tang et al., 2006). The heterogeneous terrain and diverse microhabitats likely provided shelter against climatic variations (Harrison and Noss, 2017). In contrast, the Hexi Corridor in northwestern Gansu lies within arid and semi-arid desert and grassland regions. Influenced by the aridification of the Asian interior and the uplift of the Tibetan Plateau (Liu et al., 2017), the differentiation of plant communities may have occurred in more recent geological periods, resulting in a younger average divergence time.

This southeast-to-northwest gradient in plant lineage origin times may be further attributed to regional geological and climatic gradients. The stable environmental conditions in the southeastern region have likely facilitated the persistence and continuity of ancient plant groups, while the dynamic conditions in the northwest have promoted speciation and the establishment of new lineages. Comparable patterns have been documented in the Hengduan Mountains, where the interplay of complex topography and varied climate has similarly preserved numerous ancient plant lineages (Hang, 2002). Moreover, this trend aligns with biogeographic patterns observed in other parts of the world, such as the predominance of ancient lineages in the eastern Rocky Mountains of North America versus the relatively recent origins of lineages in its arid western regions (Graham, 2011).

### 4.3 Spatial distribution of phylogenetic diversity

The analysis reveals that at the county-grid level in Gansu Province, phylogenetic diversity exhibits a spatial pattern with higher values in the southeast and lower values in the northwest. Specifically, Wenshan County in Longnan registers the highest phylogenetic diversity, while Guanghe in Linxia records the lowest. This spatial distribution is likely closely associated with the region’s geographic location, climatic conditions, and topographical complexity. At the provincial scale, the southeastern region, located in the transitional zone between the western Qinling, Hengduan Mountains, and the Qinghai–Tibet Plateau, features a complex and heterogeneous landscape that provides abundant ecological niches conducive to species evolution and diversification (Feng et al., 2014). In contrast, the northwestern Hexi Corridor is characterized by a relatively flat terrain and a homogeneous ecological environment, which may limit the development of phylogenetic diversity (Du et al., 2022). On a national level, the pattern of higher species diversity in the southeast and lower in the northwest in Gansu mirrors the overall trend of biodiversity in China. Generally, China exhibits a gradual decline in biodiversity from the southeast to the northwest, with the warmer, more humid, and topographically complex eastern and southern regions supporting higher species richness and phylogenetic diversity (Chen et al., 2022).

Moreover, the high correlation observed between species richness (SR) and phylogenetic diversity (PD) suggests that species richness partly reflects changes in phylogenetic diversity, which is consistent with previous studies (Cai et al., 2020). Species richness is often considered a key determinant of phylogenetic diversity (Webb et al., 2002). However, exceptions exist; in some arid regions, low species richness may coincide with the presence of unique evolutionary lineages that exhibit high phylogenetic distinctiveness (Arakaki et al., 2011). This indicates that the relationship between species richness and phylogenetic diversity is not always strictly positive and warrants case-specific analysis.

In Gansu Province, communities in most county-grid units—especially those in the Hexi Corridor, Loess Plateau of Central Gansu, and the Gannan Plateau—display phylogenetically overdispersed structures (SES-PD > 0), meaning that the constituent species are, on average, distantly related and have a complex evolutionary history. Such overdispersion might reflect historical roles as corridors for biotic invasion or migration, promoting the mixing of distinct phylogenetic lineages (Vellend, 2010). Similar phenomena have been observed in other regions with high environmental heterogeneity and complex historical events, such as the Mediterranean basin and the Cape Floristic Region of South Africa (Médail and Quezél, 1999; Valente et al., 2010). Conversely, communities primarily located in the eastern (Longdong) and southern (Longnan) regions tend to exhibit phylogenetic clustering (SES-PD< 0), where species are more closely related, likely due to environmental filtering that selects species with similar ecological adaptations (Loreau et al., 2001).

### 4.4 Characteristics of phylogenetic structure

Analyses of the Net Relatedness Index (NRI) and Nearest Taxon Index (NTI) at the county-grid level in Gansu Province demonstrate pronounced spatial heterogeneity in phylogenetic community assembly. In total, 55 county grid cells concurrently show NRI > 0 and NTI > 0, indicating significant phylogenetic clustering at both deep and terminal evolutionary scales. In contrast, 13 county grid cells exhibit NRI< 0 and NTI< 0, reflecting consistent phylogenetic overdispersion across phylogenetic depths. The remaining counties present discordant signals (e.g., NRI< 0 but NTI > 0), suggesting the operation of more complex or scale-dependent assembly processes. Importantly, these patterns are spatially non-random and align closely with Gansu’s major geographic regions.

Counties with simultaneous NRI > 0 and NTI > 0 are concentrated in the arid and semi-arid zones of northern and central Gansu, particularly along the Hexi Corridor and across much of the Loess Plateau. These regions are characterized by limited precipitation, high evapotranspiration, and strong temperature extremes. Such harsh abiotic environments are expected to impose strong environmental filtering, favoring lineages with shared drought- and cold-tolerant traits, thereby producing phylogenetic clustering (Webb et al., 2002, 2008). Comparable clustering driven by abiotic filtering has been documented in other dryland ecosystems, including deserts in Australia and North America, where stress-tolerant species are non-randomly assembled (Swenson et al., 2007; Jin et al., 2022).

Conversely, counties with NRI< 0 and NTI< 0 are primarily located in the humid and topographically complex Longnan mountainous region of southern Gansu, including Wen County, Diebu, Zhouqu, Wudu, and Kang County, as well as adjacent montane counties. This area experiences relatively high precipitation and pronounced habitat heterogeneity associated with deep valleys, steep slopes, and strong elevational gradients. Phylogenetic overdispersion at both broad and fine phylogenetic scales implies that competitive exclusion and niche differentiation dominate community assembly, leading to coexistence among distantly related species (Qian et al., 2024). The combination of high resource availability and environmental complexity in Longnan likely facilitates the persistence of species with contrasting evolutionary histories and ecological strategies, consistent with patterns reported from tropical rainforests and other biodiversity hotspots where niche differentiation promotes overdispersion (Webb, 2000).

Several counties, such as Lixian, Huixian, Hezuo, Xiahe, and Zhuoni, exhibit negative NRI but positive NTI and are primarily located in the transitional zones between the arid Loess Plateau and the more humid montane regions (e.g., margins of the Longnan and Gannan mountains). Overdispersion at deeper phylogenetic levels coupled with clustering near phylogenetic tips suggests that distantly related lineages co-occur at broader regional scales, whereas closely related species aggregate at finer spatial or microhabitat scales. This pattern may arise from the joint effects of historical dispersal, diversification and strong habitat heterogeneity, together with fine-scale competitive interactions among close relatives (Kraft et al., 2007). Such mixed phylogenetic structures are commonly observed in ecotonal environments where multiple biogeographic sources and environmental gradients intersect.

Comparative evidence from other regions further supports the ecological interpretation of these spatially structured patterns. For example, phylogenetic overdispersion linked to complex topography and rapid diversification has been reported in the Hengduan Mountains of southwestern China (Li et al., 2015), paralleling the overdispersed communities observed in Longnan. By contrast, the arid Hexi Corridor and Loess Plateau resemble other dryland systems where environmental filtering drives clustering of drought-adapted lineages (Swenson et al., 2007; Jin et al., 2022). Likewise, phylogenetic clustering in higher-latitude temperate forests has been associated with adaptation to cold climates (Qian and Ricklefs, 2012), indicating that although dominant abiotic filters vary among regions (e.g., drought versus cold), their net effect on promoting clustering is consistent.

Overall, the strong spatial correspondence between NRI/NTI patterns and Gansu’s major geographic divisions underscores environmental filtering as the dominant force shaping phylogenetic clustering in the arid Hexi Corridor and Loess Plateau (55 counties with NRI, NTI > 0), phylogenetic overdispersion driven by competitive exclusion and niche differentiation in the humid Longnan mountains (13 counties with NRI, NTI< 0), and mixed signals in transitional zones—provides a coherent framework for understanding how climate, topography, and habitat heterogeneity jointly shape the phylogenetic structure of plant communities across the province.

## 5 Conclusion

The Gansu flora exhibits an ancient evolutionary history, with a median lineage origin time of 19.61 Ma (early Miocene) that predates the 13.60 Ma median observed in the broader East Asian flora. This antiquity is intricately linked to Gansu’s unique geographic location at the intersection of the Tibetan, Mongolian, and Loess Plateaus, where complex topography and environmental gradients have facilitated the preservation, migration, and diversification of ancient plant clades. Additionally, the gradient of lineage origin times from southeast (older) to northwest (younger) reflects the differential impact of regional geological history and climate change. Our analyses of phylogenetic diversity (PD) and species richness (SR) across county-scale grid cells further elucidate the spatial patterns and underlying ecological processes—such as environmental filtering, competitive exclusion, and niche differentiation—that shape the phylogenetic structure of Gansu’s flora. Future research integrating fine-scale environmental variables and historical data will be essential for a more detailed understanding of the mechanisms driving the spatial distribution of phylogenetic diversity in this region.

## Data Availability

The original contributions presented in the study are included in the article/**Supplementary Material**. Further inquiries can be directed to the corresponding authors.

## Appendix

The original information on the study and more details are available in the Supplementary information.
