# Supplementary material for: Community evolution and phylogenetic structure of seed plants in Gansu, China
Source: Front Plant Sci. 2025 Dec 18;16:1693400. doi: 10.3389/fpls.2025.1693400 (PMC12756472; doi:10.3389/fpls.2025.1693400)
Supplement: Supplementary file 2 [file DataSheet2.docx]

**Supplementary information**


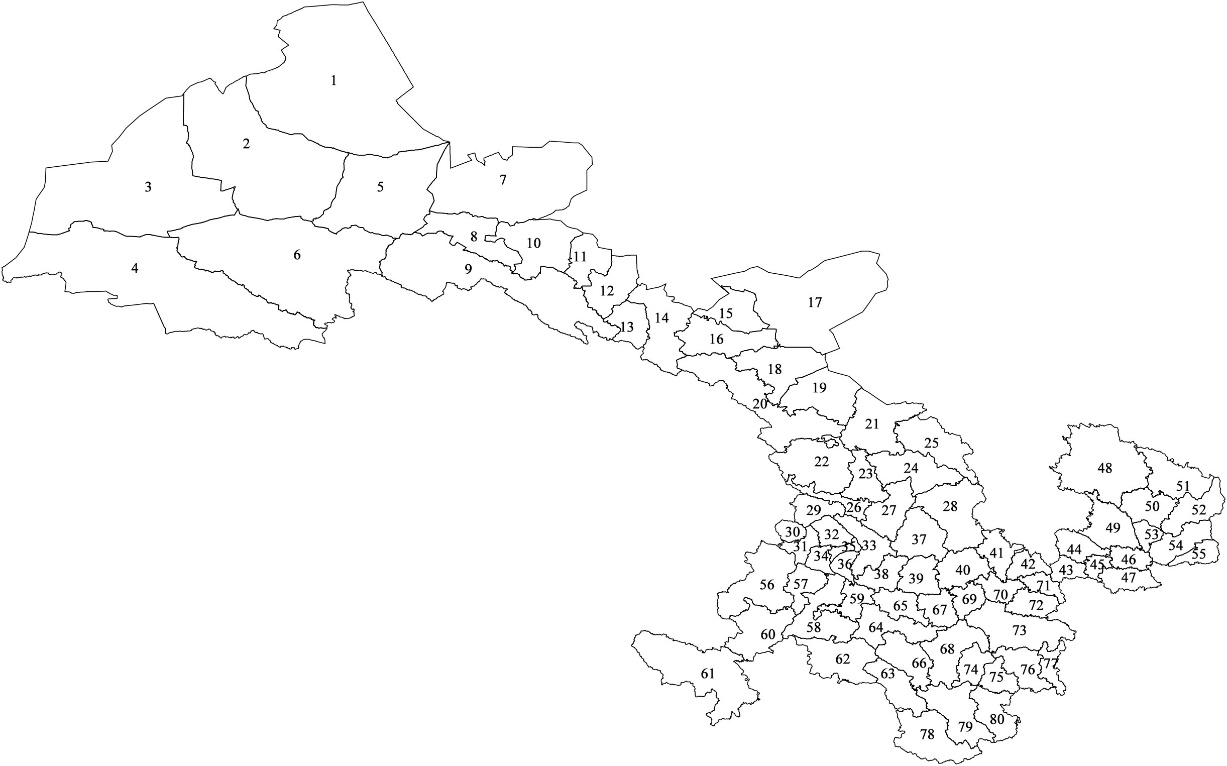


**Fig. S1** Gansu County Grid Cell. The numbers correspond with the code in Supplementary Table S1, which indicate name of each county. The map was generated using ArcGIS 10.8.

**Table S1.** Area and floristic regions for each county in Gansu. Code corresponds with the numbers in map of administrative counties (Supplementary Figure S1), which show the position of each county in Gansu.

| **Code** | **County** | **Area(km^2^)** |  |
| --- | --- | --- | --- |
| 1 | North-Subei | 38000 |  |
| 2 | Guazhou | 24100 |  |
| 3 | Dunhuang | 31200 |  |
| 4 | Akesai | 32374 |  |
| 5 | Yumen | 13389 |  |
| 6 | South-Subei | 28748 |  |
| 7 | Jinta | 18800 |  |
| 8 | Suzhou | 6321 |  |
| 9 | Sunan | 14780 |  |
| 10 | Gaotai | 6016 |  |
| 11 | Linze | 2729 |  |
| 12 | Ganzhou | 4240 |  |
| 13 | Minle | 3687 |  |
| 14 | Shandan | 9374 |  |
| 15 | Yongchang | 5867 |  |
| 16 | Jinchuan | 3060 |  |
| 17 | Minqin | 15907 |  |
| 18 | Liangzhou | 5081 |  |
| 19 | Gulang | 5103 |  |
| 20 | Tianzhu | 7147 |  |
| 21 | Jingtai | 5483 |  |
| 22 | Yongdeng | 5652 |  |
| 23 | Gaolan | 2556 |  |
| 24 | Baiyin | 4534.2 |  |
| 25 | Jingyuan | 4754.8 |  |
| 26 | Lanzhou | 1630 |  |
| 27 | Yuzhong | 3245 |  |
| 28 | Huining | 6439 |  |
| 29 | Yongjing | 1864 |  |
| 30 | Jishishan | 910 |  |
| 31 | Linxia | 1302 |  |
| 32 | Dongxiang | 1512 |  |
| 33 | Lintao | 2851 |  |
| 34 | Hezheng | 960 |  |
| 35 | Guanghe | 538 |  |
| 36 | Kangle | 1083 |  |
| 37 | Anding | 4225 |  |
| 38 | Weiyuan | 2034 |  |
| 39 | Longxi | 2657 |  |
| 40 | Tongwei | 2899 |  |
| 41 | Jingning | 2193 |  |
| 42 | Zhuanglang | 1558 |  |
| 43 | Huating | 1183 |  |
| 44 | Kongtong | 1936 |  |
| 45 | Chongxin | 852 |  |
| 46 | Jingchuan | 1409 |  |
| 47 | Lingtai | 2038 |  |
| 48 | Huanxian | 9236 |  |
| 49 | Zhenyuan | 3500 |  |
| 50 | Qingcheng | 2673 |  |
| 51 | Huachi | 3776 |  |
| 52 | Heshui | 2976 |  |
| 53 | Xifeng | 996 |  |
| 54 | Ningxian | 2633 |  |
| 55 | Zhengning | 1329 |  |
| 56 | Xiahe | 6266 |  |
| 57 | Hezuo | 2670 |  |
| 58 | Zhuoni | 4920 |  |
| 59 | Lintan | 2057 |  |
| 60 | Luqu | 4260 |  |
| 61 | Maqu | 10190 |  |
| 62 | Diebu | 5148 |  |
| 63 | Zhouqu | 3010 |  |
| 64 | Minxian | 3500 |  |
| 65 | Zhangxian | 2164 |  |
| 66 | Tanchang | 3315 |  |
| 67 | Wushan | 2011 |  |
| 68 | Lixian | 4264 |  |
| 69 | Gangu | 1573 |  |
| 70 | Qinan | 1602 |  |
| 71 | Zhangjiachuan | 1311 |  |
| 72 | Qingshui | 2012 |  |
| 73 | Tianshui | 5922 |  |
| 74 | Xihe | 1862 |  |
| 75 | Chengxian | 1678 |  |
| 76 | Huixian | 2699 |  |
| 77 | Liangdang | 1408 |  |
| 78 | Wenxian | 5002 |  |
| 79 | Wudu | 4642 |  |
| 80 | Kangxian | 2968 |  |


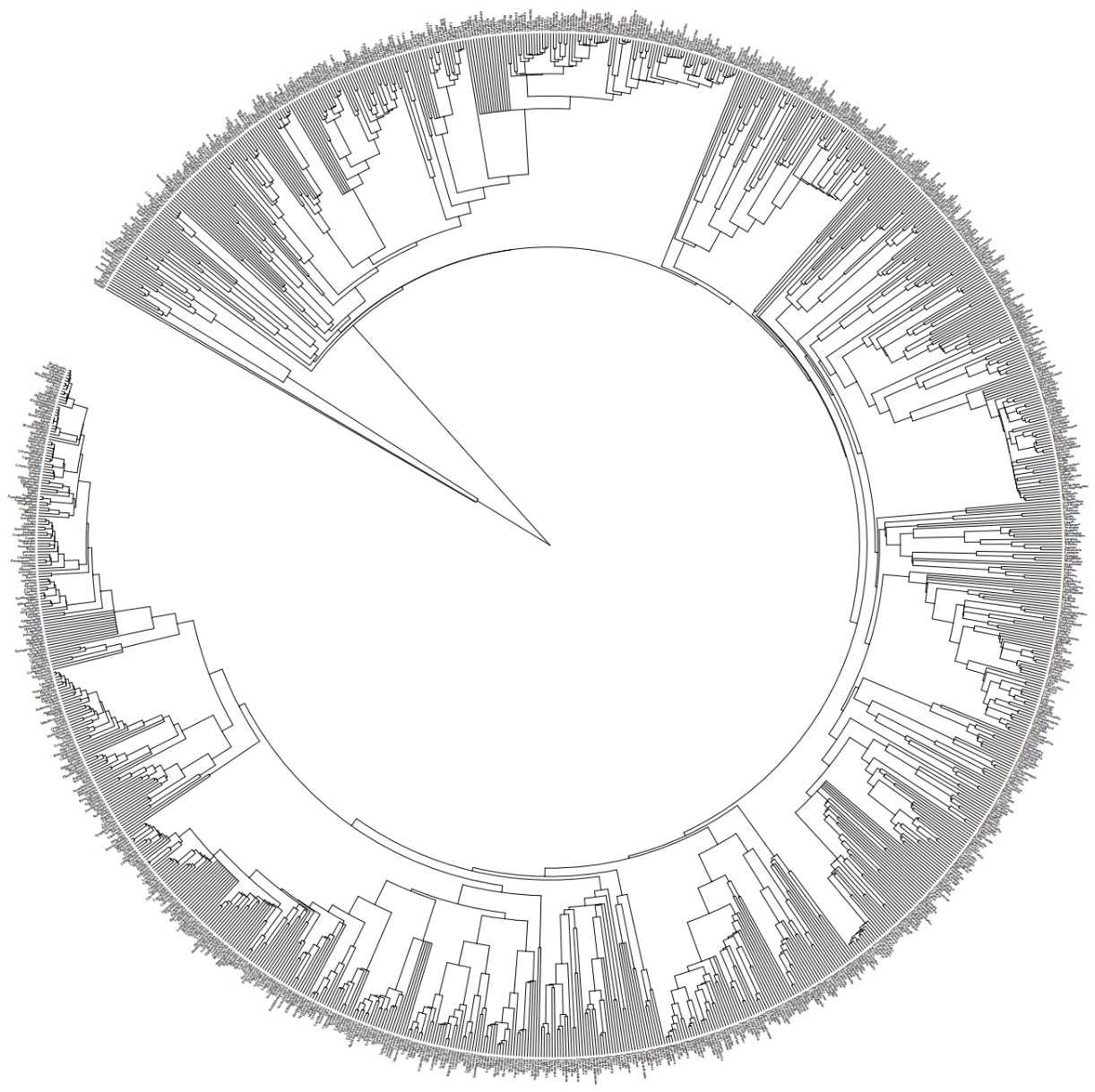


Fig. S2 Phylogenetic tree of seed plants in Gansu province at genus level


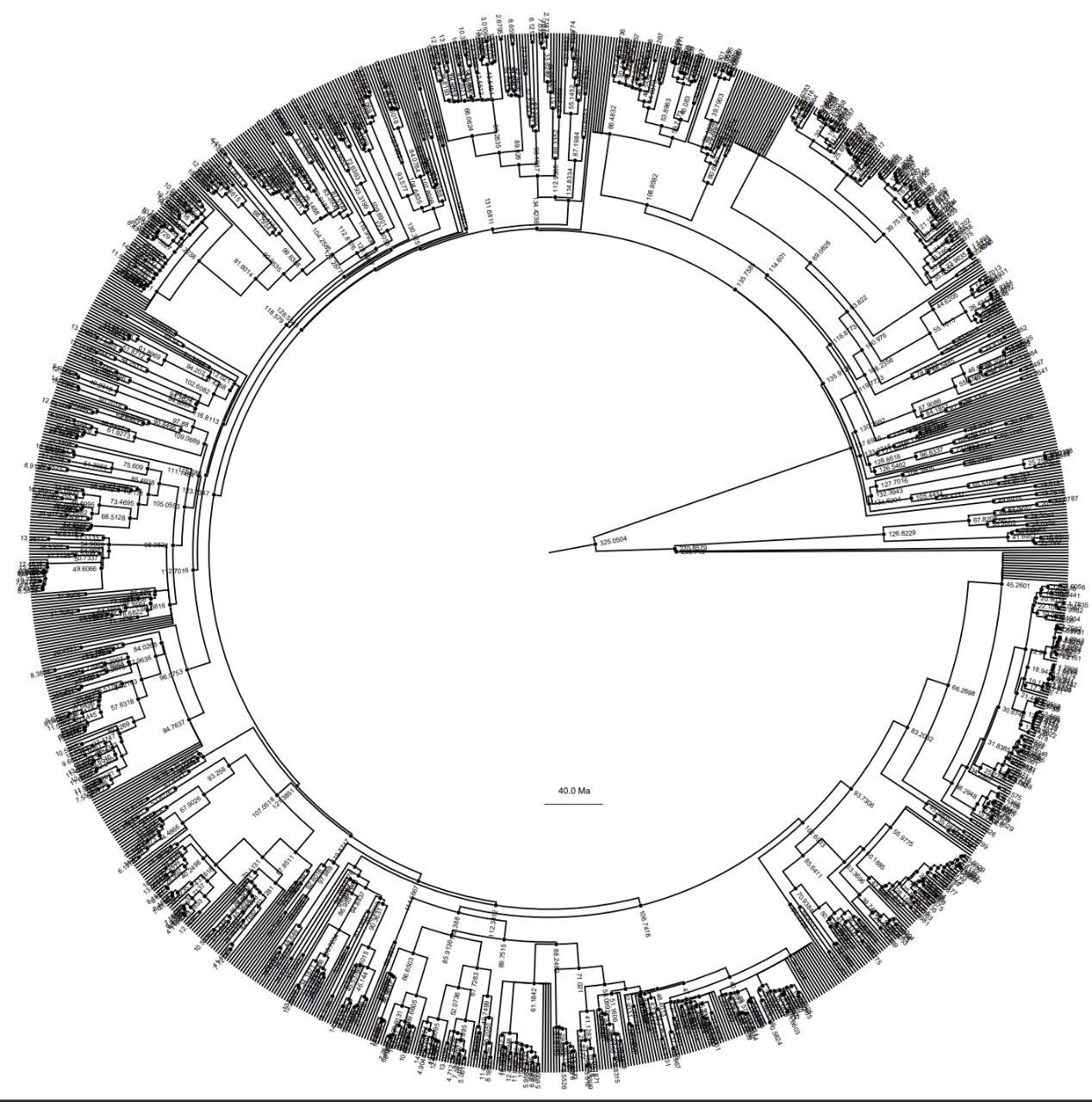
Fig. S3 Time of origin and differentiation of clades of seed plant genera in Gansu (Ma). Detailed data results are available upon request from the author.

Table S5 Mean differentiation time (MDT) and SES-MDT of county grid cells in Gansu

| Number | County Cell Grid | MDT (Ma) | SES-MDT |
| --- | --- | --- | --- |
| 1 | Wudu | 29.22895 | 0.799428 |
| 2 | Chengxian | 28.24435 | 0.003206 |
| 3 | Liangdang | 27.64175 | -0.37688 |
| 4 | Huixian | 27.51885 | -0.46213 |
| 5 | Xihe | 27.32642 | -0.51108 |
| 6 | Lixian | 27.97294 | -0.14493 |
| 7 | Kangxian | 29.75119 | 0.971993 |
| 8 | Wenxian | 29.78371 | 1.139309 |
| 9 | Tanchang | 31.82826 | 1.904885 |
| 10 | Tianshui | 27.53302 | -0.46389 |
| 11 | Qingshui | 26.66879 | -0.91467 |
| 12 | Qin'an | 26.63645 | -0.96993 |
| 13 | Gangu | 26.85737 | -0.84759 |
| 14 | Wushan | 27.57668 | -0.34787 |
| 15 | Zhangjiachuan | 26.36198 | -1.15722 |
| 16 | Hezuo | 24.97118 | -1.64795 |
| 17 | Zhouqu | 28.65292 | 0.283927 |
| 18 | Zuni | 28.14854 | -0.02159 |
| 19 | Lintan | 26.07661 | -1.06359 |
| 20 | Diebu | 27.20041 | -0.63974 |
| 21 | Xiahe | 24.24145 | -1.72575 |
| 22 | Luqu | 24.11483 | -1.73882 |
| 23 | Maqu | 23.74309 | -1.81277 |
| 24 | Linxia | 27.22308 | -0.32383 |
| 25 | Kangle | 28.98443 | 0.417376 |
| 26 | Guanghe | 24.71481 | -1.17097 |
| 27 | Yongjing | 27.96324 | -0.05173 |
| 28 | Hezheng | 29.01059 | 0.320232 |
| 29 | Dongxiang | 28.11236 | 0.027393 |
| 30 | Jishishan | 23.67602 | -1.62904 |
| 31 | Xifeng | 28.65251 | 0.237862 |
| 32 | Zhengning | 29.20682 | 0.45682 |
| 33 | Huachi | 29.02154 | 0.477763 |
| 34 | Heshui | 28.2442 | 0.061855 |
| 35 | Ningxian | 30.24344 | 0.986916 |
| 36 | Qingcheng | 29.21124 | 0.556329 |
| 37 | Zhenyuan | 29.25258 | 0.499906 |
| 38 | Huanxian | 28.54235 | 0.235797 |
| 39 | Kongdong | 27.62698 | -0.36771 |
| 40 | Jingchuan | 28.18264 | -0.01474 |
| 41 | Lingtai | 28.33211 | 0.141693 |
| 42 | Chongxin | 28.78244 | 0.221733 |
| 43 | Huating | 28.92325 | 0.477186 |
| 44 | Zhuanglang | 28.58203 | 0.174946 |
| 45 | Jingning | 28.1565 | 0.015839 |
| 46 | Anding | 24.77197 | -1.74327 |
| 47 | Tongwei | 24.97065 | -1.22462 |
| 48 | Longxi | 25.35408 | -1.00049 |
| 49 | Zhangxian | 26.08318 | -1.2735 |
| 50 | Weiyuan | 26.08682 | -0.86645 |
| 51 | Minxian | 26.44649 | -0.93464 |
| 52 | Lintao | 27.41163 | -0.33309 |
| 53 | Lanzhou | 25.53152 | -1.2615 |
| 54 | Yuzhong | 24.77266 | -1.54693 |
| 55 | Gaolan | 26.66931 | -0.63667 |
| 56 | Yongdeng | 26.35979 | -0.89491 |
| 57 | Baiyin | 24.45153 | -1.28069 |
| 58 | Huining | 26.23755 | -0.82393 |
| 59 | Jingyuan | 25.55476 | -1.23311 |
| 60 | jingtai | 22.87618 | -2.01105 |
| 61 | Liangzhou | 23.99064 | -1.6634 |
| 62 | Gulang | 23.37801 | -2.32713 |
| 63 | Minqin | 22.91071 | -2.12967 |
| 64 | Tianzhu | 23.78122 | -2.14401 |
| 65 | Jinchuan District | 21.52149 | -2.33203 |
| 66 | Yongchang | 22.86497 | -2.0886 |
| 67 | Ganzhou | 24.13636 | -1.57698 |
| 68 | Shandan | 23.13904 | -2.12893 |
| 69 | Minle | 22.87798 | -2.35342 |
| 70 | Linze | 22.88538 | -1.96735 |
| 71 | Gaotai | 22.30663 | -2.0513 |
| 72 | Sunan | 23.3764 | -2.25309 |
| 73 | Suzhou | 22.35085 | -2.54803 |
| 74 | Yumen | 22.6894 | -2.07615 |
| 75 | Dunhuang | 22.20594 | -2.34255 |
| 76 | Jinta | 21.94931 | -2.50604 |
| 77 | Guazhou | 22.39854 | -2.62214 |
| 78 | Su Bei Nan | 22.65946 | -2.60832 |
| 79 | Akse | 22.96775 | -2.47285 |
| 80 | Subei North | 21.93207 | -2.32458 |

Table S6 SR, PD, SES-PD, NRI and NTI of county grid cells in Gansu

| **County** | SR | PD | SES-PD | NTI | NRI |
| --- | --- | --- | --- | --- | --- |
| wudu | 786 | 33709.98 | -3.35 | -1.60 | -1.44 |
| chengxian | 545 | 25123.93 | -1.03 | -0.40 | -0.72 |
| liangdang | 554 | 24824.51 | 0.37 | 0.26 | 0.11 |
| huixian | 643 | 27786.11 | 0.21 | 0.74 | -0.62 |
| xihe | 478 | 21686.49 | 1.55 | 1.72 | 0.01 |
| lixian | 517 | 23355.08 | 0.80 | 1.05 | -0.28 |
| kangxian | 482 | 24083.65 | -3.35 | -1.69 | -1.39 |
| wenxian | 819 | 34244.23 | -2.24 | -0.77 | -2.71 |
| tanchang | 465 | 22947.60 | -2.33 | -1.48 | -1.65 |
| tianshui | 740 | 30831.58 | 0.23 | 0.50 | 0.59 |
| qingshui | 468 | 21182.63 | 1.87 | 1.64 | 2.10 |
| qinan | 458 | 20695.81 | 2.24 | 1.78 | 2.01 |
| gangu | 471 | 21300.65 | 1.88 | 1.51 | 2.06 |
| wushan | 498 | 22542.20 | 1.21 | 1.02 | 0.64 |
| zhangjiachuan | 453 | 20531.44 | 2.22 | 1.86 | 1.97 |
| hezuo | 411 | 19204.65 | 1.90 | 1.41 | -1.10 |
| zhouqu | 583 | 26727.74 | -1.80 | -0.43 | -1.89 |
| zhuoni | 557 | 25248.02 | -0.42 | 0.16 | -1.34 |
| lintan | 377 | 17318.73 | 3.41 | 3.07 | -2.29 |
| diebu | 623 | 27806.31 | -1.30 | -0.13 | -2.34 |
| xiahe | 288 | 13899.03 | 3.64 | 3.20 | -1.33 |
| luqu | 298 | 13348.34 | 5.48 | 4.93 | -1.96 |
| maqu | 266 | 12426.28 | 4.99 | 4.19 | -2.33 |
| linxiaxian | 165 | 9222.25 | 2.98 | 1.58 | 1.55 |
| kangle | 220 | 12235.29 | 1.18 | 1.18 | -3.76 |
| guanghe | 138 | 7825.74 | 3.36 | 2.15 | 1.54 |
| yongjing | 188 | 10591.26 | 1.90 | 2.09 | -2.81 |
| hezheng | 163 | 9329.28 | 2.34 | 2.32 | 0.87 |
| dongxinag | 163 | 9315.37 | 2.33 | 2.45 | -2.71 |
| jishishan | 147 | 8104.01 | 3.94 | 2.94 | 1.54 |
| qingyang | 244 | 13238.52 | 1.17 | 1.25 | -0.23 |
| zhenging | 274 | 15032.19 | -0.27 | -0.07 | -1.08 |
| huachi | 274 | 15011.44 | -0.23 | -0.09 | -0.41 |
| heshui | 305 | 15875.42 | 0.65 | 0.44 | -0.05 |
| ningxian | 267 | 14966.45 | -0.78 | -1.01 | -1.05 |
| qingcheng | 310 | 16414.95 | -0.24 | -0.62 | 0.73 |
| zhenyuan | 238 | 13571.51 | -0.26 | -0.68 | -0.65 |
| huanxina | 261 | 14276.72 | 0.28 | -0.11 | 0.00 |
| pingliang | 535 | 23804.40 | 1.14 | 0.77 | 1.90 |
| huating | 564 | 24676.20 | 1.45 | 1.35 | 1.68 |
| jingchuan | 260 | 13954.74 | 0.89 | 0.57 | 0.58 |
| lingtai | 251 | 13924.31 | 0.26 | -0.31 | -0.29 |
| chongxin | 245 | 13561.06 | 0.48 | 0.31 | -0.17 |
| zhuanglang | 243 | 13724.75 | -0.05 | -0.01 | -0.86 |
| jingning | 238 | 13250.59 | 0.54 | 0.01 | -0.21 |
| dingxi | 310 | 14794.53 | 3.58 | 2.48 | 2.93 |
| tongwei | 160 | 8808.08 | 3.51 | 1.94 | 1.79 |
| longxi | 147 | 8362.22 | 3.06 | 1.97 | 1.54 |
| zhangxian | 485 | 21467.99 | 2.59 | 1.60 | 2.81 |
| weiyuan | 187 | 10244.50 | 2.74 | 2.35 | 0.47 |
| minxian | 419 | 18724.94 | 3.68 | 2.55 | -1.77 |
| lintao | 200 | 10598.45 | 3.18 | 2.87 | -2.64 |
| lanzhou | 321 | 15520.99 | 2.72 | 1.86 | 1.46 |
| yuzhong | 307 | 14387.52 | 4.22 | 2.97 | 1.79 |
| gaolan | 270 | 13499.53 | 2.93 | 2.34 | 0.32 |
| yongdeng | 286 | 13837.34 | 3.73 | 3.13 | 0.99 |
| baiyin | 179 | 9522.00 | 3.57 | 2.54 | 1.14 |
| huining | 229 | 11827.56 | 3.14 | 1.69 | 1.38 |
| jingyuan | 248 | 12649.28 | 2.88 | 1.62 | 0.26 |
| jingtai | 222 | 10999.57 | 4.33 | 3.32 | 1.37 |
| wuwei | 293 | 13520.63 | 5.01 | 3.79 | 1.47 |
| gulang | 378 | 15719.68 | 6.89 | 5.42 | 1.19 |
| minqin | 287 | 12985.13 | 5.57 | 4.06 | 1.02 |
| tianzhu | 388 | 16111.68 | 6.97 | 5.55 | 1.12 |
| jinchang | 229 | 10756.43 | 5.80 | 3.96 | 1.79 |
| yongchang | 235 | 11019.60 | 5.55 | 4.26 | 1.28 |
| zhangye | 288 | 13295.54 | 4.87 | 3.91 | 1.36 |
| shandan | 369 | 15596.70 | 6.43 | 4.77 | 1.25 |
| minle | 347 | 14587.43 | 7.31 | 5.57 | 1.63 |
| linze | 247 | 11706.36 | 4.87 | 3.79 | 1.18 |
| gaotai | 247 | 11347.47 | 5.97 | 4.38 | 1.76 |
| sunan | 391 | 16260.42 | 6.60 | 5.38 | 1.14 |
| jiuquan | 292 | 13259.64 | 5.52 | 3.94 | 0.47 |
| yumen | 270 | 12503.18 | 5.20 | 3.65 | 0.83 |
| dunhuang | 277 | 12661.71 | 5.58 | 3.51 | 1.59 |
| jinta | 274 | 12278.53 | 6.11 | 4.59 | 1.01 |
| guazhou | 308 | 13412.96 | 6.37 | 4.33 | 1.52 |
| subei | 382 | 15678.08 | 7.46 | 5.88 | 0.95 |
| akesai | 376 | 15475.93 | 7.27 | 5.94 | 0.91 |
| jiayuguan | 234 | 11051.47 | 5.63 | 3.61 | 1.62 |
